# CAF-derived HGF promotes cell proliferation and drug resistance by up-regulating the c-Met/PI3K/Akt and GRP78 signalling in ovarian cancer cells

**DOI:** 10.1042/BSR20160470

**Published:** 2017-04-10

**Authors:** Wei Deying, Geng Feng, Liang Shumei, Zhao Hui, Liu Ming, Wang Hongqing

**Affiliations:** Department of Obstetrics and Gynecology, Shandong Provincial Hospital Affiliated to Shandong University, Jinan, Shandong 250021, China

**Keywords:** cancer-associated fibroblasts, cell proliferation, drug resistance, hepatocyte growth factor, ovarian cancer

## Abstract

The tumour microenvironment is a highly heterogeneous entity that plays crucial roles in cancer progression. As the most prominent stromal cell types, cancer-associated fibroblasts (CAFs) produce a variety of factors into the tumour microenvironment. In the present study, we firstly isolated CAFs from tumour tissues of the patients with ovarian cancer and demonstrated that the hepatocyte growth factor (HGF) was highly expressed in the supernatants of CAFs. CAF-derived HGF or human recombinant HGF promoted cell proliferation in human ovarian cell lines SKOV3 and HO-8910 cells. Western blotting analysis also showed that CAF-derived HGF or recombinant HGF activated c-Met/phosphoinositide 3-kinase (PI3K)/Akt and glucose-regulated protein 78 (GRP78) signalling pathways in ovarian cancer cells, and these effects could be abrogated by anti-HGF and c-Met inhibitor INCB28060. Moreover, HGF in CAF matrix attenuated paclitaxel (PAC)-caused inhibition of cell proliferation and increase in cell apoptosis through activating c-Met/PI3K/Akt and GRP78 pathways in SKOV3 and HO-8910 cells. The results *in vitro* were further validated in nude mice. These findings suggest that CAF-derived HGF plays crucial roles in cell proliferation and drug resistance in ovarian cancer cells.

## Introduction

Ovarian cancer is the seventh most common cancer in women worldwide, and it is the fifth leading cause of cancer-related deaths in women in the United States [1,2]. Epithelial ovarian cancer (EOC) that accounts for approximately 90% of all ovarian cancers is associated with high mortality rate. Current treatment of the patients with EOC usually includes surgery and cytotoxic chemotherapy [3]. In the past 20 years, the survival rate of EOC remains stable despite advances in surgical techniques and chemotherapy. One of the obstacles of chemotherapy is drug resistance. To improve the survival rate, efforts must be made to overcome the chemoresistance.

Previous studies have demonstrated that the tumour microenvironment contributes to the cancer progression and drug resistance of cancer cells [4–6]. The tumour microenvironment is composed of tumour cells, immune cells, stromal cells and the extracellular matrix (ECM) [7,8]. Cancer-associated fibroblast (CAF) is one of the most abundant stromal cell types in tumour stroma. CAFs are known to promote tumour development and progression, as well as stimulate metastasis by secreting a variety of factors [9]. Hepatocyte growth factor (HGF) is one of the growth factors secreted by CAFs, and it stimulates cell proliferation, migration and ECM invasion [10]. c-Met, as a specific receptor of HGF, is highly expressed in a number of tumour types including ovarian cancer [10,11]. The interaction of HGF and c-Met further activates downstream signalling pathways to regulate cancer progression. It has been reported that HGF/c-Met triggers downstream phosphoinositide 3-kinase (PI3K)/Akt pathway to exert the effects on cancer progression [12–14]. For instance, RNAi-mediated silencing of PI3K- and Akt-specific isoforms decrease cell proliferation, migration and invasion in ovarian cancer cells [14]. In addition, glucose-regulated protein 78 (GRP78) that is conventionally identified as an endoplasmic reticulum (ER) chaperone protein is highly induced and relocated at cell surface of various cancer cells including ovarian cancer cells [15]. Accumulated evidence has indicated that GRP78 promotes tumour growth, invasion, metastasis and drug resistance [16,17]. A recent study indicates that CAF-derived HGF inhibits paclitaxel (PAC)-induced apoptosis by up-regulating GRP78 in lung cancer [13]. In short, HGF/c-Met attracts more and more attention as a potential therapeutic target in ovarian cancer. However, whether CAF-derived HGF is involved in the cell proliferation and drug resistance in EOC has not been elucidated so far.

In the present study, we demonstrated for the first time that HGF in the tumour microenvironment promoted cell proliferation though activating PI3K/Akt and GRP78 signalling in ovarian cancer cells. Furthermore, CAF-derived HGF inhibited the PAC-induced cell apoptosis while inhibition of c-Met enhanced PAC-induced cell apoptosis. These results indicated that HGF in the CAF activated the Met/PI3K/AKT and up-regulated GRP78 expression, inducing hyposensitivity to PAC in ovarian cancer cells *in vitro* and *in vivo*.

## Materials and methods

### Isolation of normal fibroblasts and CAFs

Primary normal ovarian fibroblasts (NFs) and CAFs were isolated from the adjacent normal or tumour tissues of patients with ovarian cancer as described [18]. In brief, human ovarian cancer specimens and adjacent normal tissues, which were 50 mm far from the cancer lesions, were obtained. After several washings with sterile PBS (1.5 mM KH_2_PO_4_, 135 mM NaCl, 2.7 mM KCl and 8 mM K_2_HPO_4_, pH 7.4), tissues were cut into small squares (approximately 1 mm^3^) and incubated on an orbital shaker with 10 ml of PBS and 10 ml of 0.25% trypsin/25 mM EDTA at 37°C for 30 min. The solution containing cells in suspension was centrifuged at 1500 rpm for 5 min. NFs and CAFs were cultured in DMEM/F12 with 10% FBS and 1% penicillin/streptomycin (Thermo Fisher Scientific, Waltham, MA). Cells were maintained in a highly humidified atmosphere (80%) of 95% air/5% CO_2_ (v/v) at 37°C. Primary fibroblasts at early passages (approximately three passages) were used for the experiments to minimize dedifferentiation and modification of the original phenotype.

### Cell culture and treatment

Human ovarian carcinoma cell lines SKOV3 and HO-8910 were obtained from Cell Bank of Type Culture Collection, Chinese Academy of Science (Shanghai, China). Cells were cultured in DMEM/F12 containing 10% FBS. Cultures were maintained in a humidified atmosphere of 95% air/5% CO_2_ (v/v) at 37°C. NFs or CAFs were cultured in DMEM/F12 containing 10% FBS for 3 days. The culture medium was collected for subsequent experiments. SKOV3 or HO-8910 cells were cultured in control medium (CM) or 1:1 mixture of collected medium and fresh DMEM/F12 in the presence or absence of human anti-HGF (30 μg/ml). For recombinant HGF treatment, SKOV3 or HO-8910 cells were treated with CM or CM containing recombinant HGF (10 μg/ml) for 24–72 h. For the inhibitor study, SKOV3 or HO-8910 cells were treated with CAF matrix in the presence or absence of c-Met inhibitor INCB28060 (60 nM). For the study of drug resistance, SKOV3 or HO-8910 cells were treated with CM or CAF matrix for 24 h, and followed by the treatment of PAC (1.5 μM) in the presence or absence of c-Met inhibitor for 24 h.

### Immunofluorescence microscopy (IF)

Cells were fixed with 4% paraformaldehyde for 10 min and permeabilized with 0.1% Triton X-100 for 10 min. Cells were then blocked with 1% BSA at room temperature for 1 h. Cells were then incubated with primary antibodies (Table 1) at 4°C overnight, followed by FITC (green) or Texas Red (red)–conjugated secondary antibodies (Santa Cruz, CA) for 1 h. Slides were mounted in Fluoromount-G (Southern Biotech, Birmingham, AL; Cat#0100-01) reagent with DAPI to visualize the cell nuclei. Fluorescence images were acquired by a fluorescence microscope.

**Table 1:** The antibodies used in the present study

Name	Company	Code	Dilution ratio
Fibroblast activation protein (FAP)	Abcam	ab53066	1:50
α-Smooth muscle actin (α-SMA)	Abcam	ab7817	1:50
HGF	Abcam	ab178395	1:1000
p-Met	Santa Cruz	sc-101736	1:1000
Met	Santa Cruz	sc-514148	1:1000
p-p85	CST	4228	1:1000
p85	CST	4292	1:1000
p-AKT (Ser^473^)	CST	4060	1:2000
AKT	CST	4691	1:1000
GRP78	Abcam	ab108615	1:3000
β-actin	Santa Cruz	sc-47778	1:2000
Goat antimouse IgG-TR	Santa Cruz	sc-2781	1:150
Goat antirabbit IgG-FITC	Santa Cruz	sc-2011	1:150

### Determination of HGF concentration in cell culture medium

The levels of HGF in the culture medium of CAFs, NFs, SKOV3 and HO-8910 cells were determined by ELISA using a kit purchased from Thermo Fisher Scientific (Cat#KAC2211) according to the manufacturer’s instructions. The standard curve was established using recombinant HGF.

### Quantitative PCR

Total RNA was isolated from CAFs, NFs, SKOV3 and HO-8910 cells using TRIzol reagent (Thermo Fisher Scientific, Cat# 15596-026) and reverse transcribed with Advantage RT-for-PCR Kit (Takara, Japan; Cat# 639505). RT products were used as templates for quantitative PCR (Q-PCR). The mRNA levels of the target genes were analysed by SYBR green method with iQTM SYBR® Green Supermix (Bio–Rad, Hercules, CA; Cat# 170–3884) reagent according to the manufacturer’s instructions (*n*=3, each in triplicate). The U6 was used as an internal control for normalization. The following primers were used in the present study: HGF-F: 5′-TAGGCACTGACTCCGAACA-3′; HGF-R: 5′-AGGAGATGCAGGAGGACAT-3′; U6-F: 5′-CTCGCTTCGGCAGCACA-3′; U6-R: 5′-AACGCTTCACGAATTTGCGT-3′. The specificity of the fluorescence signal was confirmed by both melting curve analysis and agarose gel electrophoresis. The mRNA levels of target genes were determined by 2 ^–ΔΔ*C*^_T_ method.

### MTT assay

Cell viability was determined by MTT assay. SKOV3 and HO-8910 cells were seeded in 96-well plates and incubated for 24 h prior to treatment. Cells were then treated with corresponding reagents and/or drugs. Twenty microlitres of MTT was added into each well and incubated for 4 h at 37°C. After treatment, culture medium was removed and MTT formazan crystals were dissolved in 150 μl DMSO. Absorbance was measured at a wavelength of 570 nm and background absorbance was subtracted measuring at 690 nm by the use of microplate reader. All procedures were repeated at least three times.

### Annexin-V-FITC/propidium iodide staining

Annexin-V-FITC/propidium iodide staining was performed using Annexin-V-FITC/PI kit (Geneview, Cat# GK3603) according the manufacturer’s instructions. In brief, cells were cultured in six-well plates. After treatment, cells were harvested using 0.05% trypsin and washed twice with cold PBS, and then resuspended in binding buffer. Cell concentration was 1.0 × 10^6^ cells/ml. One hundred microlitres of cell solution (1.0 × 10^5^ cells) was added into a 5-ml tube. Five microlitres of Annexin-V-FITC reagent and 5 μl propidium iodide were added into each tube. Cells were gently mixed and incubated for 15 min at room temperature. Four hundred microlitres of binding buffer was then added into each tube. The samples were analysed by flow cytometry (BD FACSCalibur).

### Western blotting

Protein lysate from CAFs, NFs, SKOV3 and HO-8910 cells were prepared in Mammalian Cell Lysis/Extraction Reagent (Sigma, St. Louis, MO) supplemented with 1% Triton X-100 and 1% protease inhibitor Cocktail. Total protein concentration was determined by BCA Kit for Protein Determination (Sigma–Aldrich, Cat# BCA1). Equal amount of protein lysate were resolved by SDS/PAGE and transferred on to the PVDF membrane (Millipore, Billerica, MA) for Western blotting analysis with the corresponding primary and secondary antibodies (Table 1). ECL Western blotting detection reagents (Beyotime, Cat#P0018-1/-2) were used for protein detection. The X-ray films were scanned and bands were analysed.

### Xenograft model

BALB/c-nu nude mice were obtained from Experimental Animal Center of Shandong University. The animal study was approved by Ethics Committee of Shandong Provincial Hospital Affiliated to Shandong University. The SKOV3 cells (3.0 × 10^7^/ml) in 100 μl PBS were injected into the back of mice. The xenograft nude mice were divided into three groups: control group, HGF group and HGF + c-Met inhibitor group. Each group contains three mice. HGF (5 μg/ml) in 250 μl PBS was injected around the tumour on day 10 following tumour inoculation. For the HGF + c-Met inhibitor group, c-Met inhibitor was administered at 50 nM by intraperitoneal injection in addition to HGF treatment. Control mice were similarly treated with saline injection. Then all the mice in three groups were injected with 20 mg/kg PAC around the tumour on day 25 following tumour inoculation. Mice were terminated on day 15 PAC post treatment. Tumour size was measured every 5 days and calculated according to the formula V = L × W^2^/2. And four independent repeated experiments were taken.

### Statistical analysis

All experiments were performed for at least three times. Data are expressed as the means ± S.D. Statistical analysis was performed by two-way ANOVA. In selected experiments, a Student’s *t* test was used for paired comparisons. Statistical analysis was performed using the SPSS 17.0 for Windows software. *P*<0.05 was considered statistically significant.

## Results

### Characterization of primary CAFs and NFs

**Figure 1:**
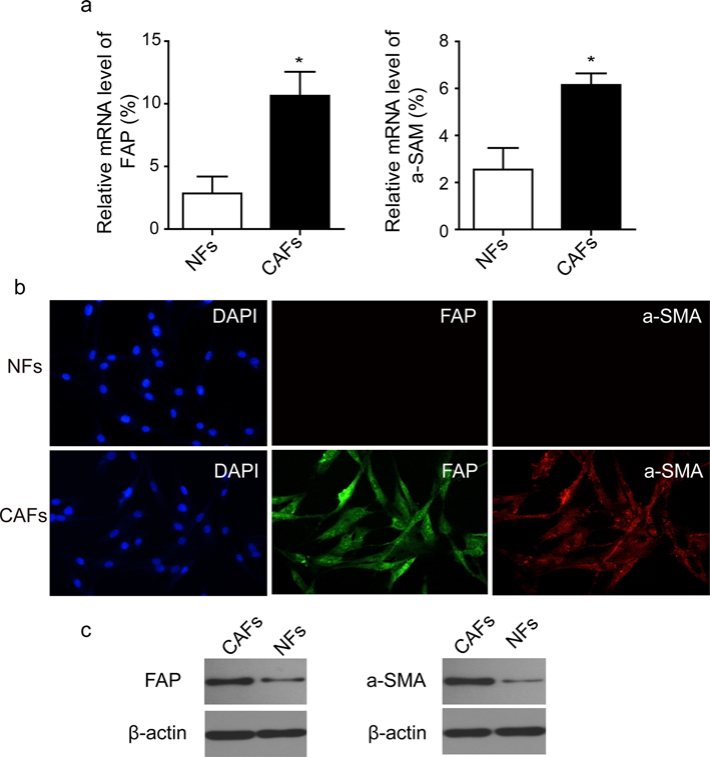
Isolation and characterization of primary CAFs and NFs (**a**) mRNA levels of α-SMA and FAP were determined by Q-PCR by using specific primers. U6 served as an internal control. (**b**) Immunofluorescence staining of FAP and α-SMA in CAFs and NFs. Primary CAFs and NFs were isolated from ovarian tumour tissue and adjacent normal tissue, respectively and cultured on the coverslips. Cells were stained with anti-FAP or anti-α-SMA antibody. Cell nuclei were visualized by DAPI. (**c**) FAP and α-SMA were analysed by Western blotting. β-actin served as a loading control. Data were representative images of three independent experiments; *, *P*<0.05.

The CAFs and NFs were isolated from ovarian tumour tissue and adjacent normal tissue, respectively. It is known that CAFs are activated fibroblasts which produce a variety of mesenchyme-specific proteins, such as FAP and α-smooth muscle actin (α-SMA) [9]. These two proteins were selected as biomarkers to test the isolation of CAFs. As shown in Figure 1a, q-PCR results showed that the mRNA levels of FAP and α-SMA in CAFs were significantly higher than that in NFs. Moreover, the immunofluorescence intensities of FAP and α-SMA were prominent in CAFs, whereas these two proteins were barely detectable in NFs (Figure 1b). Western blotting further confirmed that CAFs showed elevated expression of α-SMA and FAP compared with NFs (Figure 1c). These results showed that we successfully isolated the primary CAFs and NFs cells from ovarian tumour and adjacent normal tissue respectively.

### CAF-derived HGF promotes cell proliferation

**Figure 2:**
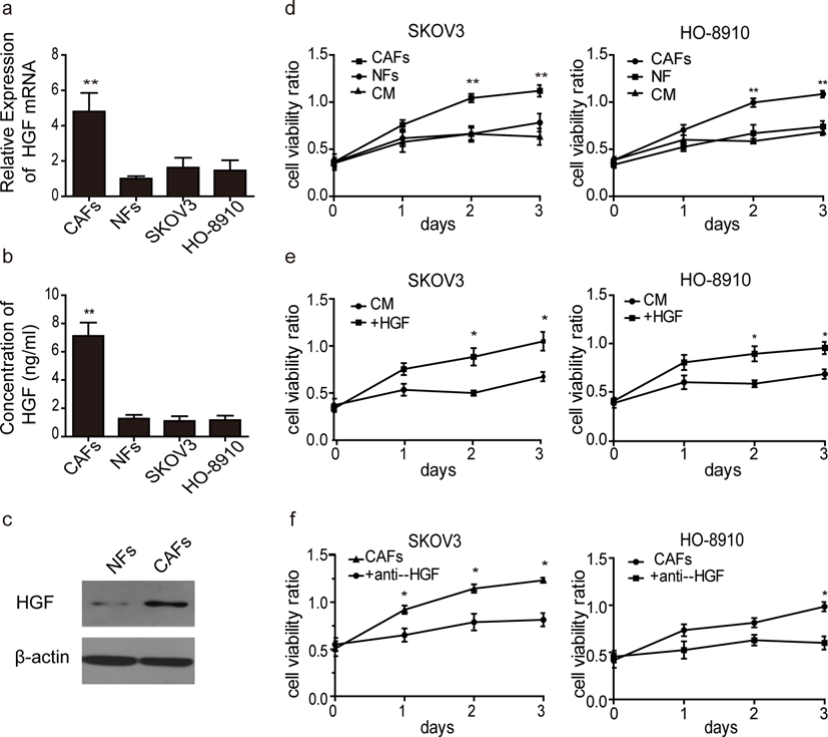
Effect of CAF matrix on cell proliferation (**a**) The mRNA levels of HGF in CAFs, NFs, SKOV3 and HO-8910 cells were determined by Q-PCR by using specific primers. U6 served as an internal control for Q-PCR; **, *P*<0.01. (**b**) The concentration of HGF secreted by CAFs, NFs, SKOV3 and HO-8910 cells were determined by ELISA. The culture medium of CAFs, NFs, SKOV3 and HO-8910 cells were collected and analysed. (**c**) The total protein level of HGF was analysed by Western blotting. β-actin served as a loading control. Data were representative images of three independent experiments. (**d**) SKOV3 or HO-8910 cells were cultured with CM, NF matrix or CAF matrix. Cell viability was monitored by MTT assay. (**e**) SKOV3 or HO-8910 cells were treated with 10 μg/ml HGF. Cell viability was monitored by MTT assay. (**f**) SKOV3 or HO-8910 cells were treated CAF matrix with or without 30 μg/ml anti-HGF. Cell viability was monitored by MTT assay. Each bar is a mean ± S.D. of three independent experiments; *, *P*<0.05; **, *P*<0.01.

It is known that tumour stroma regulates cell proliferation, morphology, survival and death. We first determined the mRNA expression and secreted protein levels of HGF in primary CAFs, NFs, SKOV3 and HO-8910 cells. As shown in Figure 2a, the results of q-PCR suggested that mRNA level of HGF was significantly higher in CAFs compared with NFs, whereas there was no difference in HGF levels among NFs, SKOV3 and HO-8910 cells. Moreover, the results of ELISA showed that the concentration of HGF in the supernatant of CAFs was approximately 7000 pg/ml (Figure 2b). In contrast, the levels of HGF in the supernatant of NFs, SKOV3 and HO-8910 cells were approximately 1000 pg/ml, which were much lower than that in CAFs. Western blotting also showed that high protein level of HGF was found in CAFs when compared with NFs (Figure 2c). Furthermore, to investigate the effect of CAF matrix on SKOV3 and HO-8910 cells proliferation, MTT assays were performed. As shown in Figure 2d, CAF matrix significantly promoted cell proliferation in SKOV3 and HO-8910 cells, whereas NF matrix and CM had no significant effect on cell proliferation. These findings suggest that HGF might be the crucial contributor in CAFs to regulate cell proliferation in ovarian cancer cells.

To further confirm the important roles of HGF, SKOV3 and HO-8910, cells were treated with human recombinant HGF (10 μg/ml). CM served as a negative control. MTT assay was performed to show the cell viability upon HGF treatment. It was apparent that HGF promoted cell proliferation (Figure 2e). In contrast, human HGF neutralizing antibody (30 μg/ml) significantly attenuated CAFs-induced cell proliferation in both SKOV3 and HO-8910 cells (Figure 2f). Collectively, these data suggested that exogenous HGF could promote cell proliferation, thus illustrating that HGF was the major contributor in CAF matrix to promote ovarian cell proliferation.

### HGF in the CAF matrix activates c-Met/PI3K/Akt pathway and induces GRP78 expression in SKOV3 and HO-8910 cells

**Figure 3:**
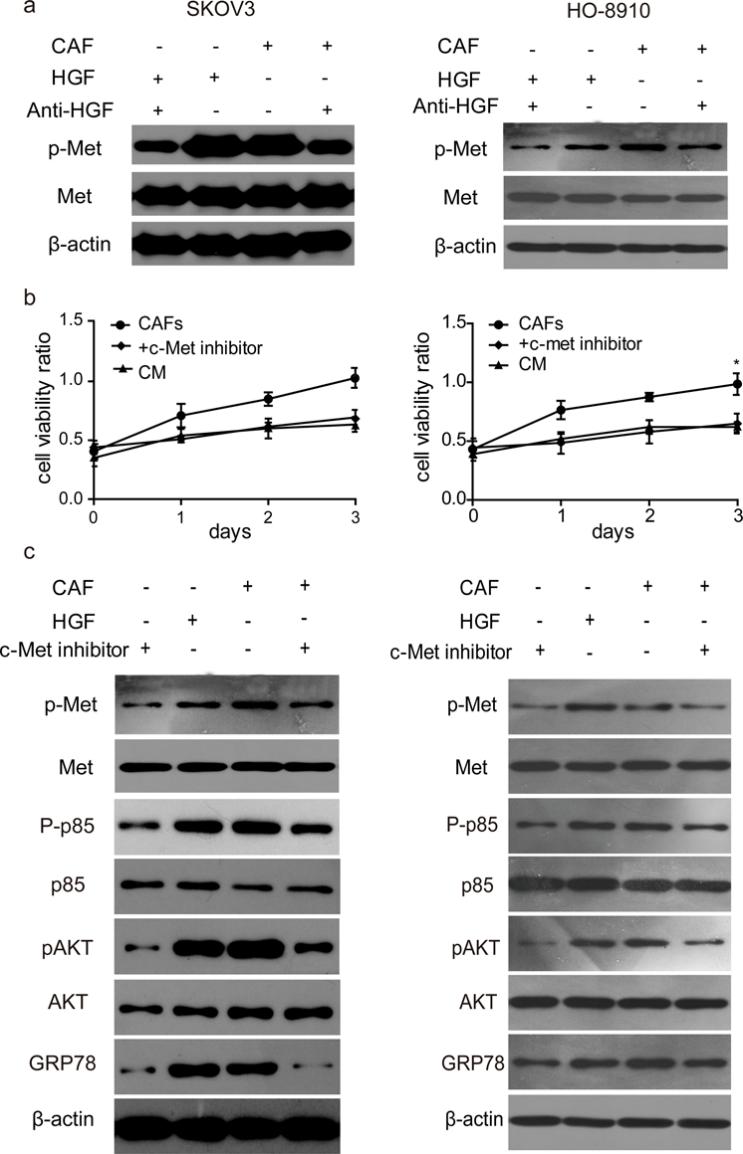
HGF in the CAF promoted the c-Met/PI3K/Akt activation and up-regulated GRP78 expression in SKOV3 and HO-8910 cells (**a**) SKOV3 or HO-8910 cells were cultured in the condition as described above. Phosphorylated c-Met and total c-Met were analysed by Western blotting. β-actin served as a loading control. Data are representative images of three independent experiments. (**b**) SKOV3 or HO-8910 cells were cultured with CAF matrix with or without c-Met inhibitor INCB28060 (60 nM). CM served as a negative control. Cell viability was monitored by MTT assay. Each bar is a mean ± S.D. of three independent experiments; *, *P*<0.05. (**c**) Phosphorylated c-Met/PI3K/Akt, total Met/PI3K/Akt and GRP78 protein level were analysed by Western blotting. β-actin served as a loading control. Data are representative images of three independent experiments.

To further investigate the downstream signalling pathways of HGF in SKOV3 and HO-8910 cells, c-Met phosphorylation was first tested by Western blotting. As expected, p-Met was increased by CAF matrix or recombinant HGF, whereas the level of total methionine did not change (Figure 3a). In addition, the activation of c-Met was abrogated by anti-HGF (Figure 3a), suggesting that CAF-derived HGF or recombinant HGF activates its receptor c-Met. We also found that c-Met inhibitor INCB28060 blocked CAF matrix-induced cell proliferation (Figure 3b), indicating that HGF in the CAF matrix induces cell proliferation through activating c-Met. Since it has been reported that HGF/c-Met activated PI3K/Akt and GRP78 pathways to regulate cancer progression, we next examined the levels of c-Met/PI3K/Akt phosphorylation and GRP78 expression in both SKOV3 and HO-8910 cells. As shown in Figure 3c, p-Met, p-PI3K/p85, p-Akt and GRP78 were increased by CAF matrix or recombinant HGF, whereas the levels of total Met, PI3K p85 and Akt did not change. This activation of c-Met/PI3K/Akt pathway and induction of GRP78 were abrogated by c-Met inhibitor INCB28060 (Figure 3c). These data indicated that HGF in the CAF matrix activated downstream c-Met/PI3K/Akt and GRP78 signalling to exert its effect in ovarian cancer cells.

### HGF in the CAF matrix decreases sensitivity to PAC in SKOV3 and HO-8910 cells

**Figure 4:**
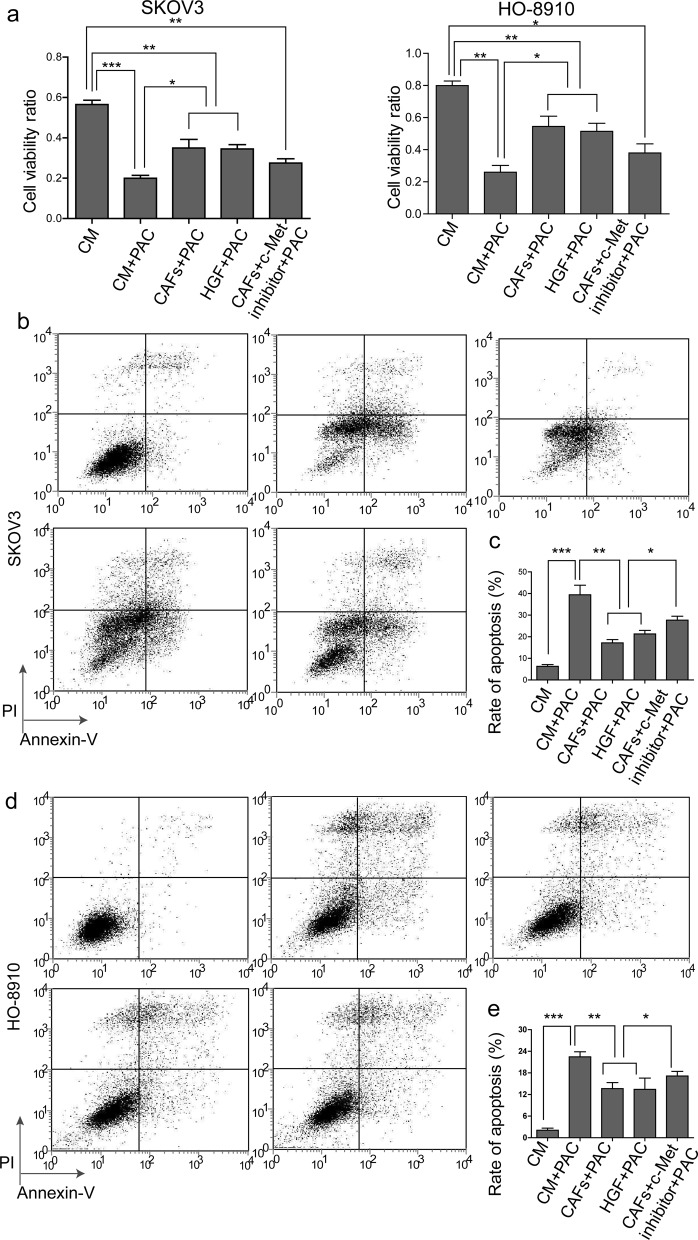
HGF in the CAF matrix decreases sensitivity to PAC in SKOV3 and HO-8910 cells (**a**) SKOV3 or HO-8910 cells were treated with CM, combination of CM and 1.5 μM PAC (CM + PAC), the combination of PAC and CAF matrix (CAFs + PAC), the combination of PAC and HGF (HGF + PAC) or the combination of PAC, CAF matrix and c-Met inhibitor INCB28060 (CAFs + c-Met inhibitor + PAC) for 24h. Cell viability was monitored by MTT assay. (**b**) SKOV3 cells were treated with CM, CM + PAC, CAFs + PAC, HGF + PAC and CAFs + c-Met inhibitor + PAC for 24 h and then cells were stained with Annexin-V-FITC and propidium iodide. The apoptotic rate was determined by flow cytometry. (**c**) Quantificative analysis of SKOV3 apoptosis. The graph shows the summarized data. (**d**) HO-8910 cells were treated with CM, CM + PAC, CAFs + PAC, HGF + PAC and CAFs + c-Met inhibitor + PAC for 24 h and then cells were stained with Annexin-V-FITC and propidium iodide. The apoptotic rate was determined by flow cytometry. (**e**) Quantificative analysis of SKOV3 apoptosis. Each bar is a mean ± S.D. of three independent experiments; *, *P*<0.05; **, *P*<0.01; ***, *P*<0.001.

PAC which is a microtubule-stabilizing reagent could induce mitotic arrest. It is a front-line agent for ovarian cancer chemotherapy in recent years. We next examined the effect of CAF-derived HGF on chemoresistance. MTT assay showed that PAC decreased cell viability by approximately 65%, whereas the combination PAC with CAF matrix or HGF caused a significant increase in cell proliferation (approximately 2-fold) when compared with the cells treated with PAC alone (Figure 4a), suggesting that HGF in the CAF matrix attenuates the effect of PAC on cell proliferation. In addition, the c-Met inhibitor slightly blocked the rescue effect of CAFs, indicating that CAFs blocked PAC-induced apoptosis through activating c-Met (Figure 4a). Moreover, Annexin-V-FITC/propidium iodide staining showed that PAC increased the cell apoptotic rate from approximately 6.5 to 39% in SKOV3 cells or from approximately 2 to 22% in HO-8910 cells respectively. In contrast, CAF matrix and HGF significantly rescued PAC-induced cell apoptosis in which the cell apoptotic rate reduced to approximately 20 and 13% in SKOV3 and HO-8910 cells respectively. But, when the c-Met inhibitor blocked the CAF signal, the rate of cell apoptosis increased both in SKOV3 and HO-8910 cells (Figure 4b–e). The quantificative data of Annexin-V-FITC/propidium iodide staining were shown in Figure 4c,e. Taken together, these data indicated that HGF in the CAF matrix induced hyposensitivity to PAC in ovarian cancer cells and the effect of HGF was mediated through c-Met.

### CAFs induced hyposensitivity to PAC by activating the c-Met/PI3K/Akt and GRP78 signalling in SKOV3 and HO-8910 cells

**Figure 5:**
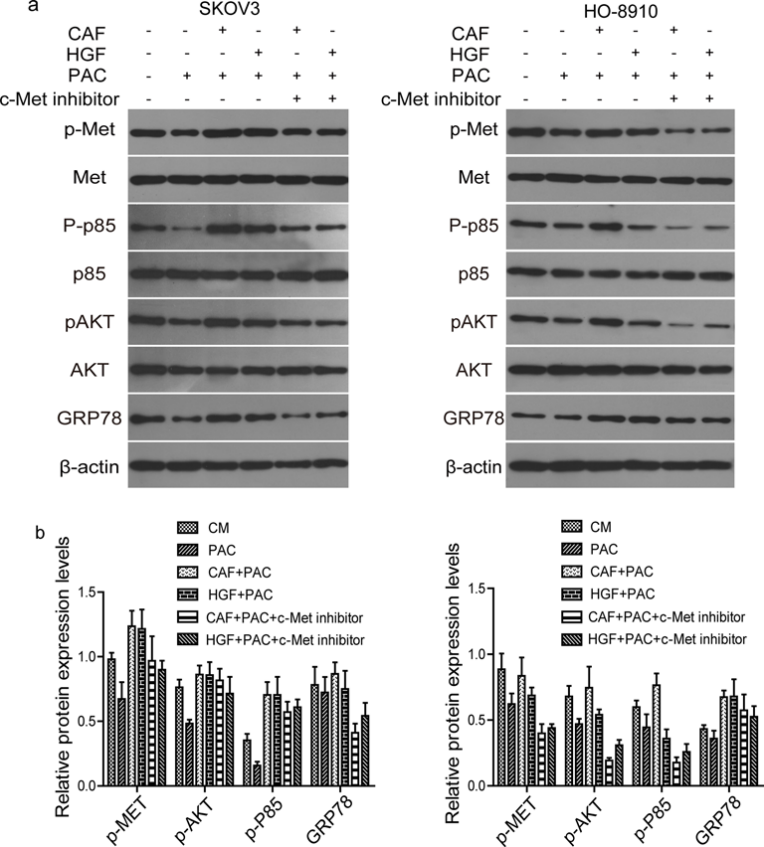
Western blotting analysis of c-Met/PI3K/Akt activation and GRP78 expression in SKOV3 and HO-8910 cells (**a**) SKOV3 or HO-8910 cells were cultured in the condition as described above. Phosphorylated c-Met/PI3K/Akt, total c-Met/PI3K/Akt and GRP78 protein level were analysed by Western blotting. β-actin served as a loading control. Data are representative images of three independent experiments. (**b**) Quantificative analysis of Western blotting data. Each bar is mean ± S.D. of three independent experiments.

In order to further study the mechanism by which CAF-derived HGF decreased sensitivity to PAC, we next examined c-Met/PI3K/Akt phosphorylation and GRP78 expression in SKOV3 and HO-8910 cells. The results of Western blotting showed that PAC significantly reduced the levels of p-Met, p-PI3K p85, p-Akt and GRP78. Whereas the matrix of CAF and HGF restored the levels of p-Met, p-PI3K p85, p-Akt and GRP78 to normal levels. Moreover, the effects of CAF and HGF were abrogated by c-Met inhibitor (Figure 5a). The quantificative data of Western blotting were shown in Figure 5b. These data indicated that HGF in CAF matrix rescued the effect of PAC through activating c-Met/PI3K/Akt and GRP78 signalling pathways.

### HGF decreased sensitivity to PAC by activating c-Met *in vivo*

**Figure 6:**
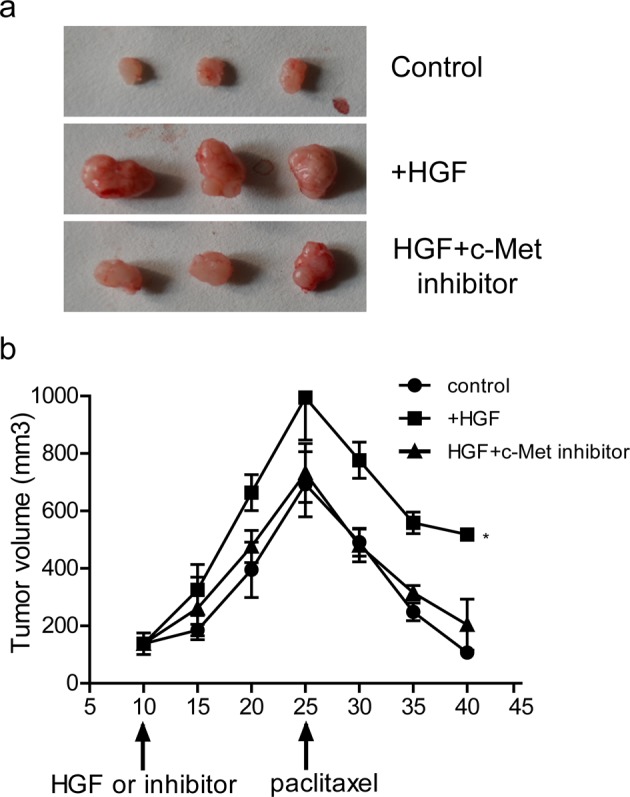
The validation in xenograft model (**a**) Images of tumour size in different groups of mice on day 40 following tumour inoculation. (**b**) Growth curves of tumour in different groups of mice; *, *P*<0.05 compared with control group.

To verify the *in vitro* results, we investigated the effect of HGF on drug resistance using *in vivo* xenograft model. The volume of tumours were calculated and recorded every 5 days until day 40, as shown in Figure 6a,b, the tumour sizes in HGF group were significantly bigger than that in control group on day 25 after tumour inoculation. When HGF pathway was blocked by c-Met inhibitor, the tumour size was reversed. We also found that the tumour volumes in three groups significantly decreased when treated with PAC after 15 days, as compared with beginning of treatment. However, the tumour volume reduction in the HGF-treated tumours was obviously less than the control and c-Met inhibitor-treated tumours. These data indicated that HGF induces drug resistance by activating c-Met pathway *in vivo*.

## Discussion

Tumour cells are heterogeneous entities whose growth is dependent on the close interaction with the ECM and with the stromal cells in the tumour microenvironment. As the key contributor in tumour microenvironment, CAFs secrete a number of factors such as cytokines, chemokines (e.g. IL-6, CXCL12), growth factors (e.g. HGF, transforming growth factor β, fibroblast growth factor) and receptors (e.g. platelet-derived growth factor receptor) [8,9]. Among these factors, IL-6 and CXCL12 have been reported to promote cell growth in prostate and breast cancer respectively. However, we found that CAF-derived HGF was the crucial contributor to stimulate cell growth and drug resistance in ovarian cancer. The important role of HGF in regulating cell growth was also confirmed by using recombinant HGF and anti-HGF antibody. These data suggested that tumour microenvironment might affect tumour growth via different mechanisms in different cancers. Moreover, it is important to investigate HGF/c-Met axis and its downstream signalling pathways within the physiological microenvironment since HGF and c-Met typically act in a paracrine manner.

c-Met is a HGF receptor that is expressed in epithelial cells. In ovarian cancer cells, c-Met is highly expressed and its overexpression is associated with adverse prognosis [10,19]. In the present study, we detected the protein levels of HGF and found CAFs produced high levels of HGF. Furthermore, HGF increased the levels of c-Met and promoted cell proliferation and drug resistance in ovarian cancer cells, consistent with the previous studies [13,20–23]. The intervention strategies of HGF/c-Met axis include (i) down-regulation of HGF or c-Met expression; (ii) disruption of HGF/c-Met interaction; (iii) inhibition of c-Met kinase activity; (iv) interference with downstream signalling pathway [11,24]. Currently, a variety of therapeutic agents targeting HGF/c-Met have been evaluated in clinical trials. These agents fall into two board categories: small molecule inhibitors and the antibodies against HGF or c-Met [11,24]. Our data also revealed that c-Met inhibitor INCB28060 abrogated CAFs-induced cell proliferation and drug resistance in ovarian cancer cells. In summary, HGF/c-Met axis is a new therapeutic target for ovarian cancer. If the underlying mechanism of HGF/c-Met axis in ovarian cancer could be better understood, the new improving therapeutic agents will be developed in the future.

As the downstream signalling pathways of HGF/c-Met, PI3K/Akt pathway is known to promote cell survival by protecting cells from apoptosis [22]. Meanwhile, a recent study has illustrated that CAF-derived chemokine, namely CCL5, promotes cisplatin resistance by the regulation of PI3K/Akt signalling pathway in ovarian cancer cells [25]. Furthermore, up-regulation of GRP78 is also associated with inhibition of cell apoptosis. Previous studies suggest that GRP78 can interact with caspase-7 or p53 to inhibit the activation of apoptosis pathway [26,27]. It can also bind to BIK and BAX to prevent cytochrome *c* release from mitochondria [28,29]. These findings indicate that activated-PI3K/Akt and GRP78 signalling pathway play an important role in cell proliferation and drug resistance. In the present study, CAF matrix or recombinant HGF caused induction of p-PI3K p85, p-Akt and GRP78 in both SKOV3 and HO-8910 cells. c-Met inhibitor blocked the activation of these two pathways and inhibited cell growth and drug resistance. It will be clinically valuable to investigate the involvement of PI3K/Akt or GRP78 inhibitors in the chemotherapy of ovarian cancer.

In summary, we have demonstrated herein tumour microenvironment, in particular CAF-derived HGF, contributed to cell proliferation and drug resistance via activating c-Met/PI3K/Akt and GRP78 signalling in ovarian cancer cells.

## Abbreviations

α-SMA: α-smooth muscle actin
AKT: AKT serine/threonine kinase 1
BAX: B cell leukemia/lymphoma 2 associated X
BIK: B cell leukemia/lymphoma 2 interacting killer
CCL5: C-C motif chemokine ligand 5
CAF: cancer-associated fibroblast
CM: control medium
CT: Cycle threshold
CXCL12: C-X-C motif chemokine ligand 12
ECM: extracellular matrix
DMEM/F12: Dulbecco's Modified Eagle Media: Nutrient Mixture F-12
EOC: epithelial ovarian cancer
FAP: fibroblast activation protein
HGF: hepatocyte growth factor
IL-6: Interleukin-6
NF: normal ovarian fibroblast
PAC: paclitaxel
PI3K: phosphoinositide 3-kinase
p85: phosphoinositide 3-kinase regulatory subunit 1
Q-PCR: quantitative PCR
RT: Reverse Transciption
RNAi: RNA interference
